# Identification of GRIN2D as a novel therapeutic target in pancreatic ductal adenocarcinoma

**DOI:** 10.1186/s40364-023-00514-4

**Published:** 2023-08-08

**Authors:** Jiatong Wang, Chi Hin Wong, Yinxin Zhu, Xiaoqiang Yao, Kelvin K C Ng, Chengzhi Zhou, Ka Fai To, Yangchao Chen

**Affiliations:** 1 School of Biomedical Sciences, Faculty of Medicine, The Chinese University of Hong Kong, Shatin NT, Hong Kong; 2 Department of Surgery, The Chinese University of Hong Kong, Shatin NT, Hong Kong; 3 State Key Laboratory of Respiratory Disease, National Clinical Research Centre for Respiratory Disease, Guangzhou Institute of Respiratory Health, The First Affiliated Hospital of Guangzhou Medical University, Guangzhou, China; 4 Department of Anatomical and Cellular Pathology, Prince of Wales Hospital, The Chinese University of Hong Kong, Shatin, Hong Kong; 5 Shenzhen Research Institute, The Chinese University of Hong Kong, Shenzhen, China

**Keywords:** Pancreatic ductal adenocarcinoma, Therapeutic target, Oncogene, NMDA receptor, GRIN2D

## Abstract

**Background:**

Pancreatic ductal adenocarcinoma (PDAC) is a devastating disease with a dismal prognosis, and despite significant advances in our understanding of its genetic drivers, like KRAS, TP53, CDKN2A, and SMAD4, effective therapies remain limited. Here, we identified a new therapeutic target GRIN2D and then explored its functions and mechanisms in PDAC progression.

**Methods:**

We performed a genome-wide RNAi screen in a PDAC xenograft model and identified GRIN2D, which encodes the GluN2D subunit of N-methyl-D-aspartate receptors (NMDARs), as a potential oncogene. Western blot, immunohistochemistry, and analysis on Gene Expression Omnibus were used for detecting the expression of GRIN2D in PDAC. Cellular experiments were conducted for exploring the functions of GRIN2D in vitro while subcutaneous and orthotopic injections were used in in vivo study. To clarify the mechanism, we used RNA sequencing and cellular experiments to identify the related signaling pathway. Cellular assays, RT-qPCR, and western blot helped identify the impacts of the NMDAR antagonist memantine.

**Results:**

We demonstrated that GRIN2D was highly expressed in PDAC cells, and further promoted oncogenic functions. Mechanistically, transcriptome profiling identified GRIN2D-regulated genes in PDAC cells. We found that GRIN2D promoted PDAC progression by activating the p38 MAPK signaling pathway and transcription factor CREB, which in turn promoted the expression of HMGA2 and IL20RB. The upregulated GRIN2D could effectively promote tumor growth and liver metastasis in PDAC. We also investigated the therapeutic potential of NMDAR antagonism in PDAC and found that memantine reduced the expression of GRIN2D and inhibited PDAC progression.

**Conclusion:**

Our results suggested that NMDA receptor GRIN2D plays important oncogenic roles in PDAC and represents a novel therapeutic target.

**Supplementary Information:**

The online version contains supplementary material available at 10.1186/s40364-023-00514-4.

## Significance

The study reveals a prominent role for GRIN2D in PDAC progression, suggesting that GRIN2D might be a novel therapeutic target for PDAC.

**Supplementary Information:**

The online version contains supplementary material available at 10.1186/s40364-023-00514-4.

## Introduction

In 2020, pancreatic cancer accounted for 495,773 new cases and 466,003 deaths globally [1]. Pancreatic ductal adenocarcinoma (PDAC), which is the most common type of pancreatic cancer, is predicted to become the second leading cancer by 2030, due to its highly aggressive malignancy and mortality [2]. Once PDAC patients are diagnosed, half of them will present with metastatic symptoms. The current standard of care for metastatic PDAC involves chemotherapy and radiotherapy, but these therapies offers limited survival benefits [3]. Consequently, there is an urgent need to identify new therapeutic targets for PDAC.

There are seven subunits in N-methyl-D-aspartate receptors (NMDARs) gene family, GRIN1, GRIN2A-D, and GRIN3A-B. These subunits regulate calcium channel by forming tetramer on the cell membrane [4]. The tetramer consists of two dimers, with obligatory subunit GRIN1 pairing randomly with GRIN2 or GRIN3. When the glycine site on GRIN1 and the glutamate site on GRIN2 or GRIN3 are occupied by specific molecules, the tetramer is activated, allowing calcium ions to enter cells [5]. Previous research on NMDAR mainly focused on the dysfunction of brain, including schizophrenia, encephalitis and epilepsy [4, 6, 7]. Notably, recent research found that aberrant expression of NMDAR subunits also occurred in cancers [8]. For example, GRIN2D is overexpressed in colorectal cancer and can promote angiogenesis [9]. Also, inhibition of GRIN2D can attenuate the tumor progression of Triple- Negative Breast Cancer (TNBC) [10]. Nevertheless, the functional and mechanistic roles of GRIN2D in PDAC are not yet fully understood.

Here, we identified GRIN2D as a potential oncogene in PDAC by genome-wide shRNA library contained mouse model. We validated that GRIN2D was overexpressed in PDAC cells and tumor tissues. It promoted cell growth, migration, invasion, colony formation, and tumor growth. We also showed that GRIN2D was involved in the epithelial-mesenchymal transition (EMT) and metastasis processes. Furthermore, we demonstrated that GRIN2D promoted PDAC progression by regulating high-mobility group A2 (HMGA2) and interleukin-20 receptor subunit beta (IL20RB) in cAMP-response-element-binding protein (CREB)/p38 Mitogen‑activated protein kinase (MAPK) signaling pathway. Treatment with the NMDAR inhibitor memantine was able to rescue the observed symptoms by suppressing GRIN2D expression and downstream signaling.

## Materials and methods

### Materials

The antibodies for the related proteins were listed below: GRIN2D (Abclonal A10080, 1:1000, for western blot), GAPDH (cell signaling, 5174 S, 1:2000, for western blot), p-CREB (cell signaling, 9198, 1:1000, for western blot), t-CREB (cell signaling, 9197T, 1:1000, for western blot), HMGA2 (cell signaling, 8179, 1:1000, for western blot), t-p38 (cell signaling, 8690T, 1:1000, for western blot), p-p38 (cell signaling, 4511T, 1:1000, for western blot), t-JNK (Cell signaling, 9252T, 1:1000, for western blot), p-JNK (cell signaling, 4668T, for western blot), p-MSK (Cell signaling, 9595T,1:1000, for western blot), t-MSK (Mybiosource, MBS8204177, 1:1000, for western blot). GRIN2D (Mybiosource, MBS9605541, 1:500, for immunohistology). Fluo-3AM (Beyotime, S1056, 1:1000, for calcium measurement). Memantine (MedChemExpress, HY-B0365A). NMDA (MedChemExpress, HY-17,551).

### Cell culture

PDAC cell line: SW1990, PANC04.03 and PANC1 were obtained from ATCC. The human pancreatic ductal epithelial (HPDE) cell line was a gift from Dr. Ming-Sound Tsao (University Health Network, Ontario Cancer Institute and Princess Margaret Hospital Site, Toronto, Canada). All cell lines were cultured under the condition as described previously [11].

### In-vivo genome-wide RNAi screening

PDAC cell line Capan2 cells were transduced with lentivirus carrying a human genome-wide shRNA library (GeneNet™ Human 50 K siRNA Library, System Biosciences). 2 × 10^6^ Capan2 cells resuspended in 10%matri- gel/PBS (infected with genome-wide shRNA library) were injected into the orthotopic pancreas of 5 nude mice. After 3–4 weeks, nude mice were sacrificed and orthotopic nodules were obtained. We then extracted total RNA from orthotopic nodules and the control group, then did RT and amplification of shRNA effectors. Finally, microarray analysis (Affymetrix HG-U133_plus_2) was performed to identify the candidate oncogenes and tumor-suppressive genes involved in PDAC development. Candidate genes whose shRNA effectors were increased by > 2 folds were considered as tumor suppressors, while candidate genes whose shRNA effectors were decreased by < -2 folds were considered as oncogenes. The data was deposited under ArrayExpress database with accession number E-MTAB-9074.

### Immunohistochemistry staining

The clinical samples were obtained from patients who underwent pancreatic resection at the Prince of Wales Hospital, Hong Kong, with written informed consent obtained from all patients recruited and the study was conducted in accordance with the ethical guidelines and approval of the Joint CUHK-NTEC Clinical Research Ethics Committee, following the Declaration of Helsinki. The method of obtaining PDAC tissue specimens was described before [12]. Tumor samples from nude mice were obtained after euthanasia.

Paraffin embedding was performed on all specimens, and the resulting slides were subjected to deparaffinization in xylene and rehydration in ethanol with a gradient ratio. Antigen retrieval was achieved by incubating the slides in sodium citrate buffer at 85 °C for 20 min. The blocking, primary antibody incubation, secondary antibody incubation, and DAB staining procedures were conducted according to the instructions provided with the Rabbit-specific HRP/DAB (ABC) Detection IHC Kit purchased from Abcam (ab64261).

### Bioinformatic analysis

Paired comparison of tumor and normal tissues was performed using the GSE15471 dataset from the Gene Expression Omnibus (GEO) database, sample size was 39 pairs. While unpaired comparison was based on the GSE16515 dataset, tumor sample size was 36 and normal sample size was 16. Mutation analysis was performed using the cBioportal website (https://www.cbioportal.org). TCGA database (https://www.cancer.gov/about-nci/organization/ccg/research/structural-genomics/tcga) was used to investigate the expression of GRIN2D in various cancers. Correlation analysis was conducted using data from the Xenabrowser database (https://xenabrowser.net), and the Human Cancer Metastasis Database (HCMDB) (https://hcmdb.i-sanger.com/index) was used to compare the expression of genes in primary and metastatic tumors.

### qRT-PCR

RNA of the cells and tumor was extracted by Trizol. Quality and quantity of RNA was measured by nanodrop (Biorad). Reverse transcription was performed with Mir-X miRNA First-Strand Synthesis Kit (Takara). The cDNA was used for the mRNA detection via MM Core RT04 - ABI QuantStudio 7 (QS7) Flex Real Time PCR System.

### Functional assay

Migration assay was performed in 12 well plates with migration inserts. Transfected and drug treated cells (50 thousand) in 125 μl of complete DMEM were incubated at 37 °C in 5% CO_2_ incubator overnight. The next day, inserts were removed and the complete DMEM was replaced by DMEM without FBS. Migration was monitored by microscope (Nikon) at the origin magnification ×40 in every timepoints.

Cell proliferation assay was performed in a 96-well plate, 1500 cells were cultured in 100 μl complete medium per well. The absorbance was recorded after one hour incubation with the mixture of 90% complete medium and 10% CCK8 reagent.

For the invasion assay, 10% matrix gel in blank DMEM was firstly added to the chamber for 3 h. Then, the suspended cells were cultured in the chambers with the blank DMEM, the chambers were settled in the 24 well plate contained complete DMEM. After 72 h, the chambers were used for crystal violet staining.

For colony formation assay, it was performed in 6 well plate. 2 × 10^3^ cells were seeded per well, and the colonies were stained after 15 days.

### RNA sequencing

After transfection with siGRIN2D for 72 h, total RNA was extracted from SW1990 and PANC04.03 cells using Trizol. The RNA samples were used for library preparation, next-generation sequencing, and bioinformatic analysis at BGI Hong Kong Co., Limited. Sequencing data are available in the NCBI Sequence Read Archive (SRA) under accession number PRJNA894301.

### Calcium influx measurement

Cells were seeded in confocal dish the day before measurement. Before the measurement, cells were incubated with Fluo-3AM for an hour. Olympus Fluoview ver. 4.2 was used for recording fluorescence intensity. 100μM NMDA was used for inducing the increased calcium influx.

### Chromatin immunoprecipitation

Chromatin immunoprecipitation (Ch- IP) was performed based on the prediction of transcription factors with Jaspar database (https://jaspar.genereg.net). The predicted binding sequence of IL20RB: CTGTGAGTTCATT, start from 1729 and end to 1741 with the score of 7.5256176. The predicted binding sequence of HMGA2: TGTACACGTTACCA, start from 93 and end to 106 with the score of 9.483109.

Ch- IP was performed with High-sensitivity Ch- IP Kit (Abcam, ab185913). The sheared chromatin for the reaction was from SW1990, PANC04.03 and PANC1 cells. DNA extraction was performed in accordance with the protocol, and binding interactions between genes were assessed using RT-qPCR.

### In vivo subcutaneous injection and orthotopic injection

Female BALB/c nude mice aged 4 to 6 weeks were obtained from Laboratory Animal Services Centre of the Chinese University of Hong Kong (Shatin, Hong Kong). Animal handling and experimental procedures were approved by the Animal Experimental Ethics Committee of the institute. For tumor growth assay, 7 × 10^5^ cells were resuspended in 1×PBS with 20% Matrigel. The mixture was injected subcutaneously into the left flank and orthotopically into the head of pancreas. After tumor formation, tumor growth was monitored every two or three days and tumor volume was measured using calipers with the equation: volume = (length×width^2^)/2. Tumors were collected for RNA extraction and IHC staining.

### Drug treatment

Memantine was dissolved in sterile deionized water and added to complete cell culture medium for the treatment. The medium was refreshed every 48 h to maintain the concentration of memantine.

### Statistical analysis

Statistical analysis was performed with GraphPad Prism 7. Statistically significant was considered when P value (two-sided) was less than 0.05.

## Results

### GRIN2D was identified as an oncogene in PDAC

**Fig. 1:**
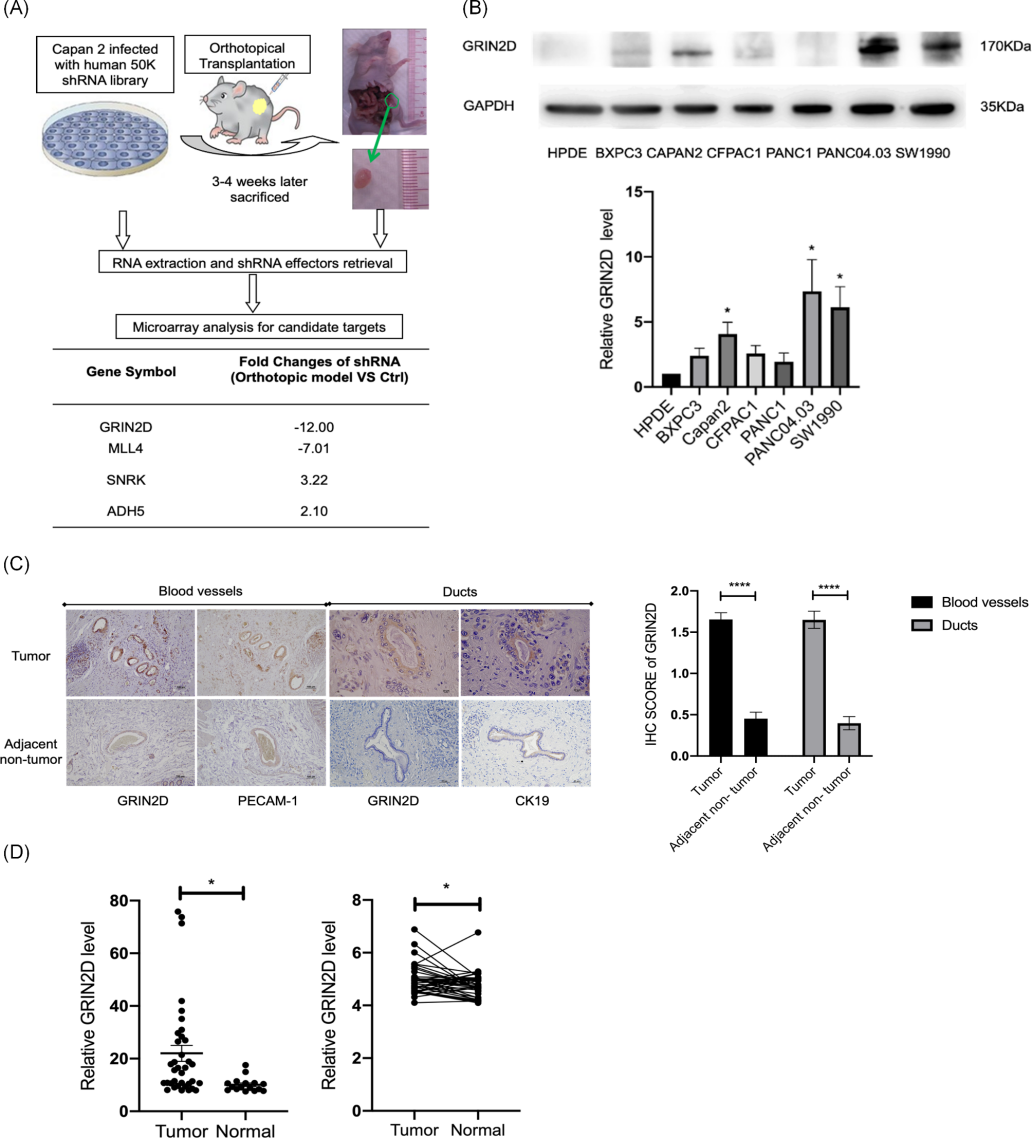
GRIN2D was identified as an oncogene in PDAC. (**A**). Workflow of in-vivo genome-wide RNAi screening. (**B**). GRIN2D was upregulated in PDAC cells, as compared to non-tumor HPDE cells. (**C**). IHC staining of GRIN2D in blood vessels and ducts with the respective markers PECAM-1 and CK19. GRIN2D was upregulated in PDAC tumor tissues, compared to adjacent non- tumor tissues. (**D**). GRIN2D expression was increased in PDAC tumors, as compared to normal, from two independent samples cohort GSE16515 and GSE15471. Data are from at least three independent experiments. Mean ± SD. *, P < 0.05; **, P < 0.01; ***, P < 0.001

To identify PDAC-associated genes as well as potential therapeutic targets, we employed a lentiviral-based shRNA library targeting the entire human genome to enable genome-wide loss-of-function analysis in PDAC cell line Capan2. Candidate genes were identified based on at least a 2-fold change in shRNA effectors in orthotopic nodules compared to control cells. The fold change of N-methyl D-aspartate 2D (GRIN2D) was − 12, it was the highest absolute value in the screening which could be identified as an oncogene in PDAC (Fig. 1A).

To investigate the expression of GRIN2D in PDAC cells, we performed western blot first and found it was overexpressed in several PDAC cell lines compared to non-tumor cell HPDE (Fig. 1B). To examine the GRIN2D expression in PDAC patients, we performed immunohistochemical (IHC) staining on primary tumors and adjacent non-tumor tissues from 72 PDAC patients. We observed that GRIN2D expression was increased in the ducts and blood vessels of tumor tissues compared to adjacent non-tumor tissues (Fig. 1C). We also analyzed publicly available datasets (GSE16515 and GSE15471) and found that GRIN2D was significantly upregulated in PDAC tumor tissues compared to non-tumor tissues (Fig. 1D). Furthermore, pan-cancer analysis using The Cancer Genome Atlas (TCGA) datasets revealed that GRIN2D was upregulated in multiple cancers (Fig. S1A). Notably, GRIN2D expression was higher in metastatic tumors than in primary tumors based on analysis from HCMED database (Fig. S1B). We also observed that the mutation rate of GRIN2D in PDAC patients was only 0.5%, indicating that mutations are unlikely to be the cause of GRIN2D overexpression (Fig. S1C). Taken together, our findings suggest that the upregulation of GRIN2D may play a critical role in PDAC development and progression.

In addition to GRIN2D, we investigated the potential importance of other NMDAR subunits in PDAC. GRIN2D was identified as the only one subunit in NMDAR gene family which highly upregulated in primary tumor tissue than the normal tissues in both of GEO datasets GSE15471 and GSE16515(Fig. S1D and E).

### GRIN2D promoted PDAC tumorigenesis in vitro

**Fig. 2:**
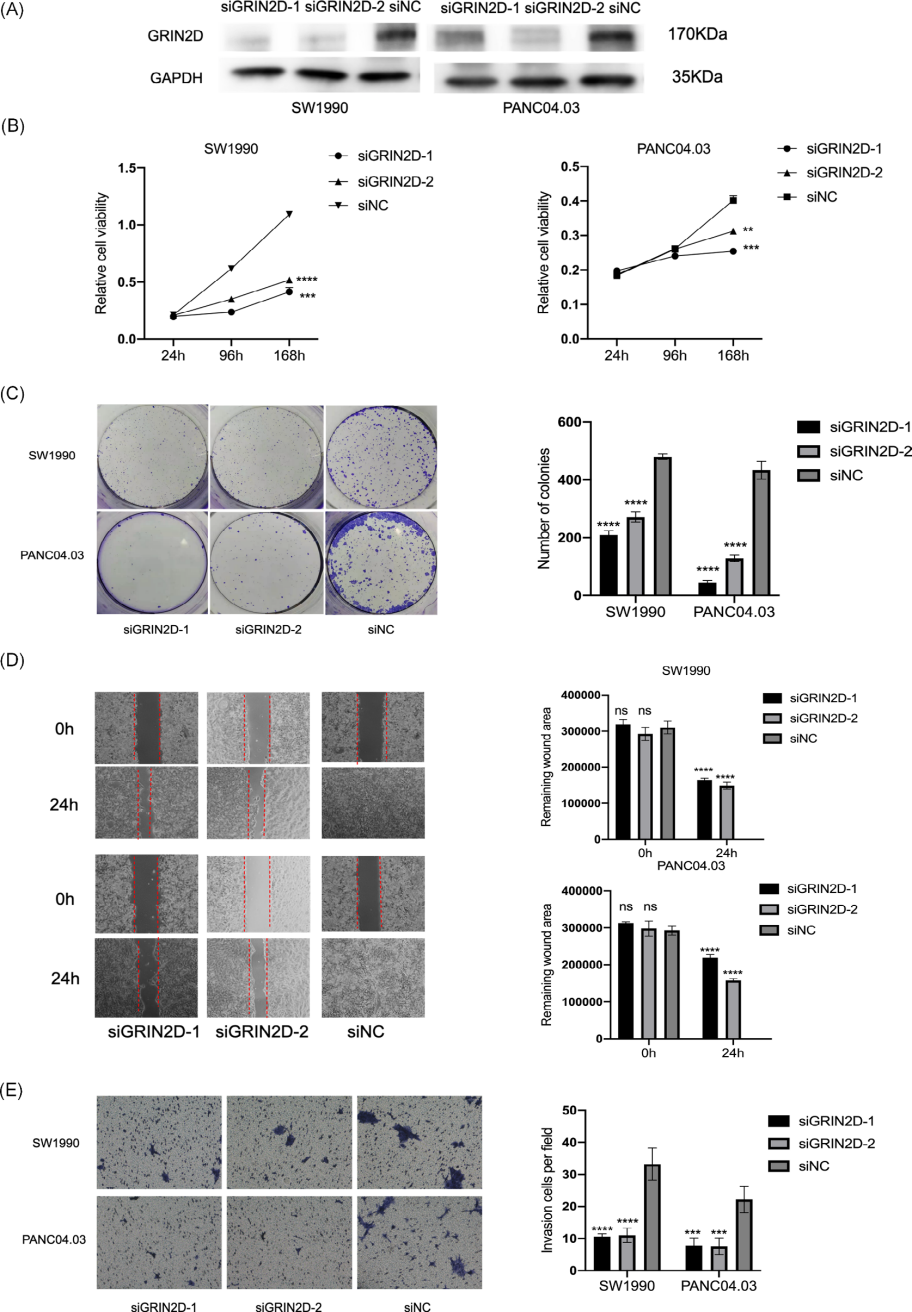
GRIN2D drove tumorigenic functions in PDAC cells. (**A**). GRIN2D was knocked down by siRNAs in SW1990 and PANC04.03 cells. (**B**). Knockdown of GRIN2D inhibited cell growth in PDAC cells. (**C**). Knockdown of GRIN2D inhibited colony formation in PDAC cells. (**D**). Knockdown of GRIN2D inhibited cell migration in PDAC cells. (**E**). Knockdown of GRIN2D inhibited cell invasion in PDAC cells. Cells in the colony formation assay and invasion assay were stained by crystal violet. Data are from at least three independent experiments. Mean ± SD. *, P < 0.05; **, P < 0.01; ***, P < 0.001

To investigate the functional roles of GRIN2D in PDAC progression, we utilized siRNAs to knockdown of GRIN2D in PDAC cells (Fig. 2A), followed by examining the effects on PDAC progression. We found that knockdown of GRIN2D could inhibit the PDAC cell growth (Fig. 2B) and significantly reduced colony formation after 14 days of incubation (Fig. 2C). The decreased GRIN2D expression could also impair PDAC cell migration (Fig. 2D) and reduced the invasion ability of the two cell lines (Fig. 2E). Then, we overexpressed GRIN2D in PANC1 cells (Figure. S2A) and found that it significantly promoted cell growth, colony formation, migration, and invasion in PANC1 cells (Fig. S2B to E).

### GRIN2D promote PDAC progression by p38 MAPK signaling

To investigate the molecular mechanism underlying the regulation of PDAC progression by GRIN2D, we used siRNA to knockdown of GRIN2D in SW1990 and PANC04.03 cells, followed by transcriptome profiling. We identified the differentially expressed genes upon GRIN2D knockdown and performed the Kyoto Encyclopedia of Genes and Genomes (KEGG) pathway analysis. The results showed that the GRIN2D- regulated genes were involved in multiple biological pathways, including the MAPK, TNF, and VEGF signaling pathways (Fig. S3A). Given that MAPK signaling is frequently reported to play critical roles in regulating calcium channels, we examined whether knockdown of GRIN2D could reduce calcium influx in PDAC cells. As we expected, the reduced level of GRIN2D can inhibit the calcium influx in cells. (Fig. S3B). Since MAPK signaling was the most significantly enriched pathway regulated by GRIN2D, we further investigated this pathway in detail.

**Fig. 3:**
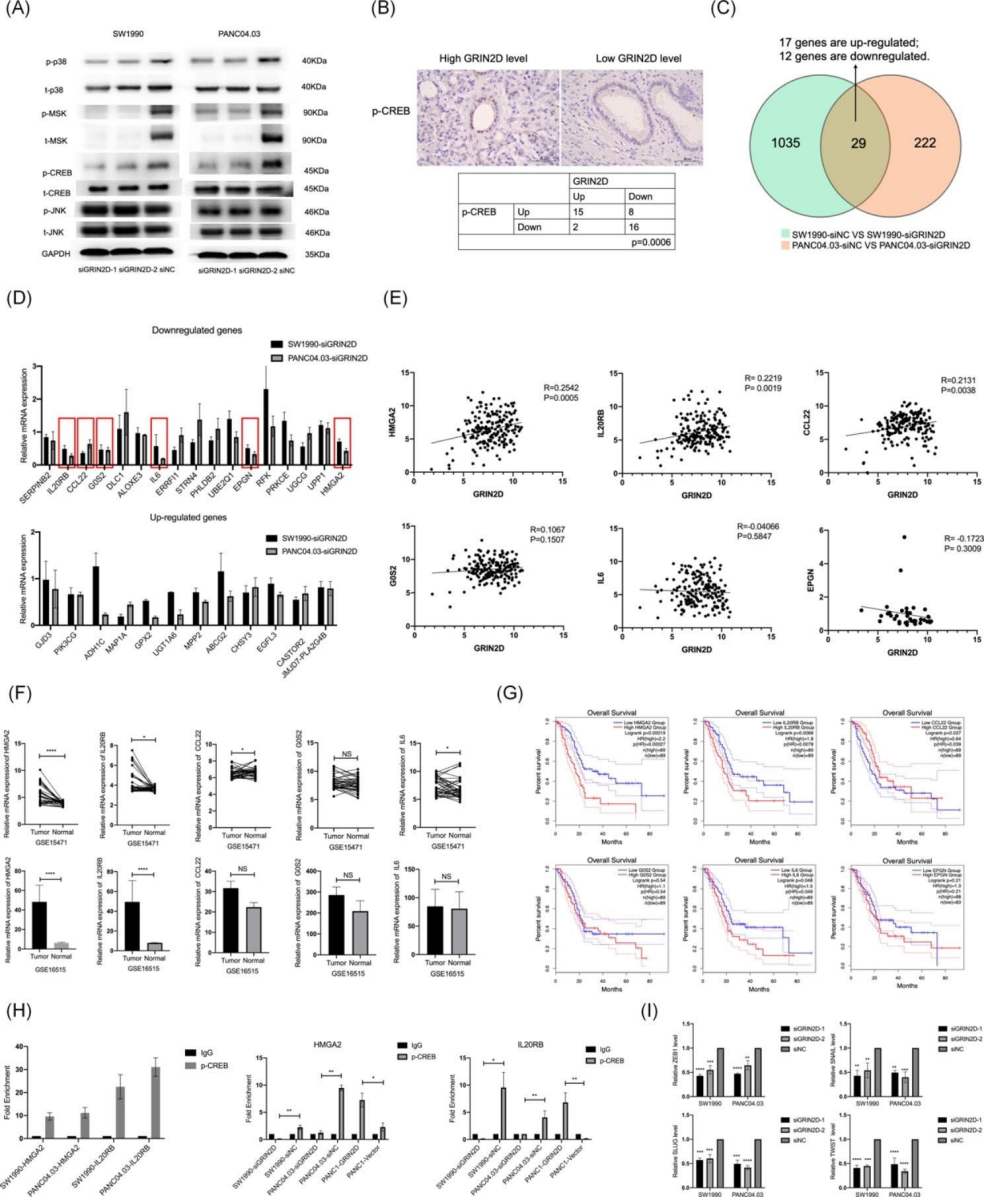
GRIN2D promotes PDAC progression by CREB/ p38 MAPK signaling pathway. (**A**). p38, MSK, and CREB were dephosphorylated after knockdown of GRIN2D in SW1990 and PANC04.03 cells. (**B**). Clinical correlation between GRIN2D and p-CREB levels in PDAC primary tumors. (**C**). Amount of common differential genes in SW1990 and PANC04.03 cells after knockdown of GRIN2D. (**D**). Expression of differential genes after knockdown of GRIN2D. (**E**). Correlation between GRIN2D and IL20RB, CCL22, G5S2, IL6, EPGN, and HMGA2 expression in PDAC tumors. (**F**). IL20RB, CCL22, G5S2, IL6, EPGN, and HMGA2 expression in tumor and non- tumor groups from two independent samples cohort GSE16515 and GSE15471. (**G**). Survival analysis of IL20RB, CCL22, G5S2, IL6, EPGN, and HMGA2 in PDAC patients. (**H**). Phosphorylated CREB binding to HMGA2 and IL20RB in SW1990 and PANC04.03 cells, as revealed by ChIP assay. (**I**). EMT markers ZEB1, SNAIL, SLUG, and TWIST were downregulated after knockdown of GRIN2D in SW1990 and PANC04.03 cells. Data are from at least three independent experiments. Mean ± SD. *, P < 0.05; **, P < 0.01; ***, P < 0.001

Based on the KEGG pathway database of MAPK signaling, we hypothesized that GRIN2D promotes PDAC progression through the JNK and p38 MAPK signaling pathways (Fig. S3C). To validate our hypothesis, we performed the western blot after the knockdown of GRIN2D in SW1990 and PANC04.03 cells. We found that the knockdown of GRIN2D could inhibit the phosphorylation of p38 and its downstream effectors MSK and CREB, without affecting the phosphorylation of JNK (Fig. 3A). These results suggested that GRIN2D promoted PDAC progression by regulating p38 MAPK signaling. To investigate the clinical significance of GRIN2D in regulating p38 MAPK signaling, we measured the expression of phosphorylated CREB in primary PDAC tumors. We found a positive correlation between phosphorylated CREB and GRIN2D expression in primary tumors (Fig. 3B). Collectively, our results suggest that GRIN2D plays a critical role in promoting PDAC progression through the regulation of the p38 MAPK signaling pathway.

To further understand the mechanism how GRIN2D regulates PDAC progression via the p38 MAPK signaling pathway, we examined the results of transcriptome profiling and identified 29 differentially expressed genes in both of two SW1990 and PANC04.03 (Fig. 3C). We further validated that IL20RB, CCL22, G5S2, IL6, EPGN, and HMGA2 were downregulated by GRIN2D knockdown (Fig. 3D). By examining their expression in Xenabrowser, we found that HMGA2 and IL20RB were positively correlated with GRIN2D expression and highly expressed in PDAC tumor tissues (Fig. 3E and **F**). Moreover, their high expression was associated with poor survival in PDAC patients (Fig. 3G). Then, we investigated whether HMGA2 and IL20RB were regulated under GRIN2D- p38 MAPK signaling pathway. Bioinformatic analysis using the JASPAR transcription factor database revealed the CREB binding site on HMGA2 and IL20RB. Furthermore, the ChIP assay confirmed the binding of p-CREB with HMGA2 and IL20RB (Fig. 3H).

Additionally, we examined the effects of the knockdown of HMGA2 and IL20RB on the oncogenic functions of GRIN2D-overexpressed PANC-1 cells and found that their knockdown attenuated these functions (Fig. S4A to D). Notably, HMGA2 and IL20RB were more highly expressed in metastatic tumors than in primary tumors, consistent with the trend observed for GRIN2D expression (Fig. S5A and B). In conclusion, our results suggest that HMGA2 and IL20RB are downstream genes of GRIN2D in the CREB/p38 MAPK pathway and involve in PDAC progression.

Given that HMGA2 has been reported to promote EMT and that knockdown of GRIN2D reduces the expression of HMGA2, we hypothesized that GRIN2D might be involved in regulating EMT in PDAC. To test this hypothesis, we examined the expression of EMT markers in SW1990 and PANC04.03 cells after knockdown of GRIN2D. We found that knockdown of GRIN2D could downregulate the expression of ZEB1, SNAIL, SLUG, and TWIST, indicating that GRIN2D promotes EMT in PDAC (Fig. 3I).

### GRIN2D promoted tumor growth and liver metastasisin vivo

**Fig. 4:**
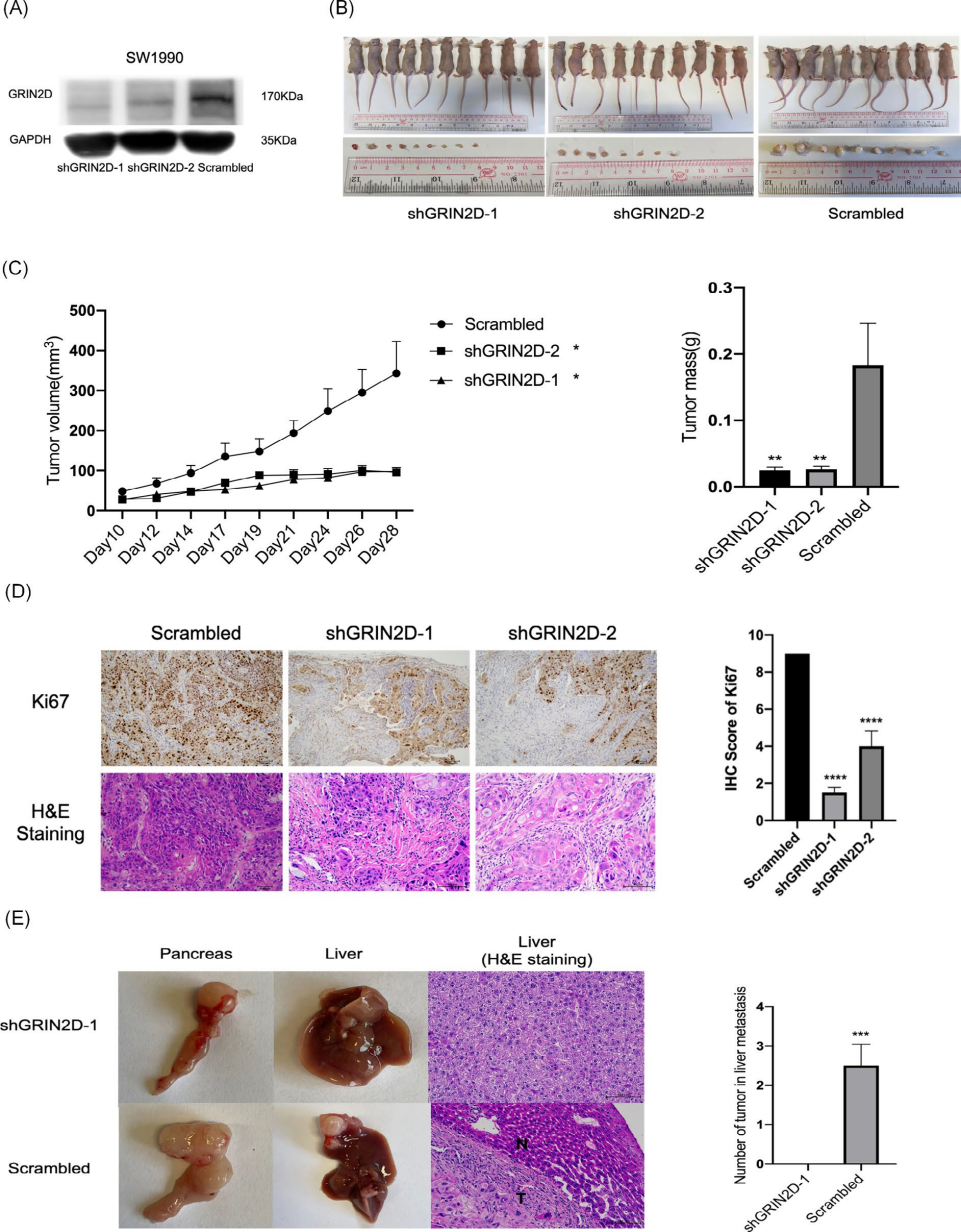
GRIN2D promoted tumor growth and liver metastasis in PDAC. (**A**). GRIN2D was knocked down by stable lentiviral shRNA system in SW1990 cells. (**B**). Knockdown of GRIN2D inhibited tumor growth in SW1990 cells. Photographs of mice xenograft at day 28 after knockdown of GRIN2D in SW1990 cells. (**C**). Tumor volume and tumor mass of xenograft mice after knockdown of GRIN2D. (**D**). IHC staining of Ki67, H&E Staining in mice subcutaneous tumors after knockdown of GRIN2D. (**E**). Knockdown of GRIN2D inhibited liver metastasis in SW1990 cells

To characterize the role of GRIN2D in vivo, we constructed shGRIN2D SW1990 cells (Fig. 4A), then established xenograft mice models and performed subcutaneous injection (Fig. 4B). Knockdown of GRIN2D could significantly inhibit tumor growth and tumor mass (Fig. 4C). IHC staining on the tissues indicated that knockdown of GRIN2D could reduce ability of cell proliferation, as detected by the marker Ki67(Fig. 4D). Overexpression of GRIN2D in PANC1 cell could promote tumor growth (Fig. S6A and B). We then examined the HMGA2 and IL20RB expression in mice PDAC xenograft with knockdown of GRIN2D and found that knockdown of GRIN2D in vivo could also reduce the expression of HMGA2 and IL20RB (Fig. S6C). Since in vitro studies revealed that GRIN2D promoted PDAC migration and invasion (Fig. 2D and **E**). To investigate the role of GRIN2D in PDAC metastasis in vivo, we constructed metastatic mouse models through orthotopic injection of SW1990 shGRIN2D cells into the pancreas. We found that knockdown of GRIN2D significantly inhibited PDAC metastasis to the liver (Fig. 4E). Taken together, our results suggest that GRIN2D plays a crucial role in promoting PDAC tumor growth and liver metastasis.

### Memantine is a potential drug for PDAC therapy

**Fig. 5:**
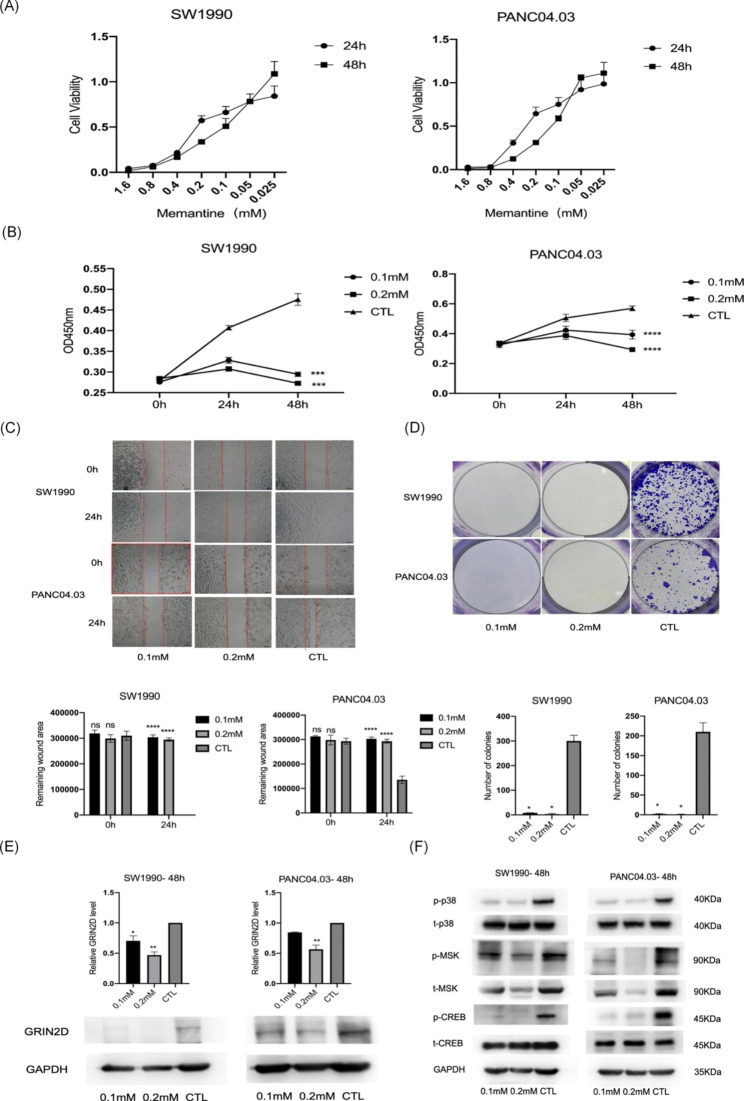
Memantine is a potential drug for PDAC therapy. (**A**). Dose-response curve of memantine in PDAC cells. (**B**). Cell growth was inhibited in memantine treated PDAC cells, as revealed by CCk8 cell viability assay. (**C**). Cell migration was inhibited in memantine treated PDAC cells, as revealed by wound healing cell migration assay. (**D**). Clonogenic ability was inhibited in memantine treated PDAC cells, the cells in the colony formation assay were stained by crystal violet. (**E**). Level of GRIN2D decreased in memantine treated PDAC cells. (**F**). p38, MSK, and CREB in p38 MAPK signaling pathway were dephosphorylated after treatment of memantine. Data are from at least three independent experiments. Mean ± SD. *, P < 0.05; **, P < 0.01; ***, P < 0.001

Given that GRIN2D is the subunit of NMDAR family, we next examined the effects of NMDAR antagonist memantine in PDAC cells. We firstly constructed a dose-response curve in PDAC cells and determined 0.1mM and 0.2mM for cells at 24 and 48 h incubation (Fig. 5A). We found that memantine inhibited cell proliferation, cell migration, and colony formation at these concentrations (Fig. 5B to **D**). Additionally, memantine effectively reduced the expression of GRIN2D (Fig. 5E) and downregulated p38 MAPK signaling pathway, as evidenced by the decreased phosphorylation of p38, MSK, and CREB (Fig. 5F). In summary, memantine reduced GRIN2D levels and inhibited phosphorylated CREB/p38 MAPK signaling to alleviate PDAC progression.

## Discussion

In this study, we identified the role of GRIN2D in promoting cancerous functions and confirmed the mechanism in PDAC progression. Additionally, we suggested that the NMDAR antagonist memantine could be a potential therapy for PDAC.

To investigate the roles of GRIN2D in PDAC, we first examined its expression in PDAC tumors. We found that GRIN2D was overexpressed in PDAC primary tumor and even higher in metastatic tumor, which is consistently observed using PDAC tumors from HCMDB database. Although many RNA sequencing datasets in public databases indicated that GRIN2D was aberrantly expressed in cancers, its specific role in promoting cancer progression remained unclear. In a study on colorectal cancer, GRIN2D was detected to be overexpressed in patients’ tissues at both mRNA and protein levels, and the function on promoting angiogenesis was identified in cellular assay [9]. In breast cancer, the research on miR-129-1-3p illustrated that it acted as a tumor repressor and bound with GRIN2D, but no further detailed work on GRIN2D in breast cancer functionally and mechanically [10]. Thus, our study was the first to identify the role of GRIN2D in cancer progression at deeper level.

Our study demonstrated that the upregulated expression of GRIN2D in PDAC cells and tumor tissues played a significant role in cell proliferation, migration, invasion and metastasis both in vitro and in vivo. RNA sequencing revealed that GRIN2D promoted PDAC progression by upregulating HMGA2 and IL20RB under CREB/ p38 MAPK signaling pathway. Our study also found that NMDAR antagonist memantine could inhibit the level of GRIN2D and its downstream signaling pathway in PDAC.

Therefore, targeting GRIN2D could be a promising approach for PDAC therapy. As a subunit of NMDAR, GRIN2D is known to be involved in developmental and epileptic encephalopathies (DEEs) by forming the variants and changing channel open probability [13]. In colorectal cancer, GRIN2D overexpression was observed in blood vessels of tumor tissues, promoting angiogenesis [9]. Similarly, in our study, we observed overexpression of GRIN2D in both blood vessels and ducts of PDAC tumors as the respective markers PECAM-1 and CK19 showed [14, 15].

Based on transcriptome profiling of GRIN2D knockdown, we found that many GRIN2D-regulated genes were involved in the MAPK signaling pathway, which has been shown to mediate calcium signaling in the nervous system [16]. Study in osteoblasts demonstrated that the activated calcium channels could increase the calcium influx, which subsequently activated ERK1/2 and p38 MAPK signaling [17]. In our study, the knockdown of GRIN2D significantly reduced calcium influx in PDAC cells, and most of the GRIN2D-regulated genes were involved in c-Jun NH2-terminal kinase (JNK) and p38 MAPK signaling, with some focusing on downstream p38 MAPK signaling. Knockdown of GRIN2D inhibited phosphorylation of CREB, an activated transcription factor that recruits transcription coactivators to promote the expression of target genes involved in cellular activities [18]. CREB can activate the signal transducer and activator of STAT3 pathway for the tumorigenesis in pancreatic cancer [19]. We suggested that GRIN2D promoted PDAC tumorigenesis by increasing calcium influx, which in turn activated p38 MAPK signaling and the transcriptional function of CREB. A previous study using a mouse model of pancreatic neuroendocrine tumorigenesis (PNET) suggested that interstitial fluid pressure led to invasion progression by activating NMDAR and its downstream MEK-MAPK and Ca^2+^/calmodulin-dependent kinases (CaMK) effectors, ultimately caused the activation of CREB [20]. However, in that study, the aberrant expression of the NMDAR subunit was GRIN2B, not GRIN2D. We did not detected overexpression of GRIN2B in tumor tissues in our bioinformatic analysis. Overall, our findings suggested that targeting GRIN2D could be a potential therapeutic strategy for PDAC treatment, as it plays a significant role in promoting PDAC tumorigenesis through the activation of p38 MAPK signaling and transcription factor CREB.

We identified HMGA2 and IL20RA as downstream targets under the GRIN2D- CREB pathway in PDAC. HMGA2 has been shown to play a significant role in promoting EMT and metastasis in several studies [21–23]. For instance, HMGA2 has been found to promote the expression of EMT marker SNAIL via CREB in liver cancer [22]. In our study, we demonstrated that GRIN2D can also promote EMT, as knockdown of GRIN2D inhibited the expression of several EMT markers including SNAIL, SLUG, ZEB1, and TWIST. EMT is a critical driver of metastasis, as it regulates transcription factors like SNAIL, TWIST, and ZEB, which in turn promotes the conversion from epithelial to mesenchymal state and enhances the ability of cancer cells to metastasize to other sites [24]. Our in vivo study further confirmed that GRIN2D could promote liver metastasis. These results suggest that GRIN2D promotes EMT and liver metastasis in PDAC via the CREB-HMGA2 pathway.

In addition to HMGA2, we found that GRIN2D could promote the IL20RB expression. IL20RB could cooperate with interleukin-20 receptor subunit alpha (IL20RA) to form interleukin-20-receptor I complex (IL-20-RI) for binding with cytokines, such as IL-19, IL-20 and IL-24. IL20R cytokines is expressed in both immunity and epithelium related cells [25]. Previous studies have shown that IL20RB is involved in promoting oncogenic functions in papillary renal cell carcinoma [26], while a retrospective meta-analysis on 466 PDAC patients demonstrated the prognostic value of IL20RB, but the detailed function of IL20RB in PDAC progression remains unknown [27]. In current study, we found that IL20RB was also potentially involved in metastasis progression in pancreatic cancer and other cancers based on the analysis in HCMDB.

To explore potential therapeutic targets in PDAC, we investigated the possibility of targeting GRIN2D. In previous research on neurological diseases, numerous inhibitors have been developed to treat NMDAR-related disorders. Here, we used the NMDAR uncompetitive inhibitor memantine which has been approved for moderate-to-severe Alzheimer’s disease treatment [28]. Memantine has been validated to induce apoptosis and autophagy by decreasing the metabolism of lung cancer cells [29]. Besides, memantine could cause apoptosis and inhibit cell cycle in prostate cancer [30]. In our study, we demonstrated that memantine could inhibit cell proliferation, migration, and colony formation by reducing the expression of GRIN2D and inhibiting the p38 MAPK signaling pathway in PDAC. Therefore, memantine could potentially be used as a therapeutic agent for targeting GRIN2D in PDAC. Additionally, we propose that other NMDAR antagonists that target GRIN2D could be tested for PDAC treatment. For example, MK-801, another NMDAR antagonist, has been shown to inhibit cell proliferation and invasion in pancreatic cancer and hepatocellular carcinoma [31, 32]. Collectively, these studies indicate the potential anti-cancer potency of NMDAR antagonists. Further detailed studies will be conducted to develop drugs targeting GRIN2D for PDAC treatment.

Based on our results, it appears that GRIN2D is involved in regulating calcium influx and calcium-related signaling pathways. Glutamate is the activator of NMDAR and is formed from glutamine through the catalysis of GLS or GLS2 [33]. However, glutamine is the primary nutrient for the growth of cancer cells which involved in the tricarboxylic acid (TCA) cycle, nucleotide and fatty acid biosynthesis, and redox balance [34]. Excessive consumption of glutamine may result in a decrease in the production of glutamate, leading to the under-activation of NMDAR. In fact, the obligatory NMDAR subunit GRIN1 was downregulated in tumor tissues based on the analysis of GEO database. This analysis indicates that GRIN2D might play a role in maintaining calcium influx in PDAC patients. Calcium ion (Ca^2+^) is reported to regulate various processes, such as cell proliferation [35], invasion [36], metastasis [37], angiogenesis [38], transcription [39] and apoptosis [40]. Therefore, it is essential to maintain calcium ion homeostasis. The key factors for regulating the homeostasis are Ca^2+^ channels, pumps and exchangers [41, 42]. These factors can also be regarded as therapeutic targets for cancers [43]. Previously, some Ca^2+^ channel receptors have been identified as potential therapeutic targets, such as TRPV6 in ER-negative breast cancer [44] and CaSR in bone metastasis [45]. Our study has expanded the research on calcium channel receptors in cancer area.

## Conclusion

Our study revealed the overexpression of GRIN2D in PDAC tissues and cells. It could promote cancerous functions both in vitro and in vivo. RNA sequencing analysis supported the involvement of the CREB/p38 MAPK signaling pathway in GRIN2D-mediated PDAC progression. Furthermore, we demonstrated the inhibitory effect of NMDAR antagonist memantine on GRIN2D expression and downstream signaling pathways. These findings highlighted the potential of GRIN2D as a clinically relevant biomarker for PDAC and a promising therapeutic target for novel treatment strategies.

### Electronic supplementary material

Below is the link to the electronic supplementary material.

**Supplementary Material 1: Figure S1**: (A). GRIN2D expression was upregulated in various cancers, according to samples from TCGA database. (B). Level of GRIN2D in primary tumor and metastasis tumor based on HCMDB. (C). Mutation of GRIN2D in PDAC and other cancer based on cBioPortal database. (D). Expression of NMDAR subunits GRIN2A, GRIN2B, GRIN2C, GRIN3A, and GRIN3B in PDAC primary tumors and normal tissues from patients in cohort of GSE15471. (E). Expression of NMDAR subunits GRIN2A, GRIN2B, GRIN2C, GRIN3A, and GRIN3B in PDAC primary tumors and normal tissues from patients in cohorts of GSE16515

**Supplementary Material 2: Figure S2** (A). GRIN2D was overexpressed in PANC1 cells. (B). Overexpression of GRIN2D promoted cell growth in PANC1 cells. (C). Overexpression of GRIN2D promoted colony formation in PANC1 cells. (D). Overexpression of GRIN2D promoted cell migration in PANC1 cells. (E). Overexpression of GRIN2D promoted cell invasion in PANC1 cells. Cells in the colony formation assay and invasion assay were stained by crystal violet. Data are from at least three independent experiments. Mean ± SD. *, P < 0.05; **, P < 0.01; ***, P < 0.001

**Supplementary Material 3: Figure S3**: (A). KEGG pathway enrichment analysis of the GRIN2D-regulated genes. (B). Calcium influx measurement after knockdown of GRIN2D in PDAC cells. (C). Map of differential genes in JNK and p38 MAPK signaling pathway

**Supplementary Material 4: Figure S4**: (A). Knockdown of HMGA2 and IL20RB in GRIN2D- overexpressed PANC1 cells inhibited cell growth. (B). Knockdown of HMGA2 and IL20RB in GRIN2D- overexpressed PANC1 cells inhibited cell invasion. (C). Knockdown of HMGA2 and IL20RB in GRIN2D- overexpressed PANC1 cells inhibited colony formation. (D). Knockdown of HMGA2 and IL20RB in GRIN2D- overexpressed PANC1 cells inhibited cell migration. Data are from at least three independent experiments. Mean ± SD. *, P < 0.05; **, P < 0.01; ***, P < 0.001

**Supplementary Material 5: Figure S5** (A). Level of HMGA2 in primary tumor and metastasis tumor based on HCMDB. (B). Level of IL20RB in primary tumor and metastasis tumor based on HCMDB

**Supplementary Material 6: Figure S6**: (A). GRIN2D overexpression promoted tumor growth in PANC-1 cells. Photographs of mice xenograft at day 15 after overexpression of GRIN2D in PANC-1 cells. (B). Tumor volume and tumor mass of xenograft mice increased after overexpression of GRIN2D. (C). HMGA2 and IL20RB were downregulated in GRIN2D knocked down tissues. Data are from at least three independent experiments. Mean ± SD. *, P < 0.05; **, P < 0.01; ***, P < 0.001

Supplementary Material 7

## Data Availability

In-vivo genome-wide RNAi screening data was deposited under ArrayExpress database with accession number E-MTAB-9074; RNA sequencing data are available in the NCBI Sequence Read Archive (SRA) under accession number PRJNA894301.

## List of abbreviations

CaMK: Calmodulin-dependent kinases
ChIP: Chromatin immunoprecipitation
CREB: CAMP-response-element-binding protein
DEEs: Developmental and epileptic encephalopathies
EMT: Epithelial-mesenchymal transition
GEO: Gene Expression Omnibus
HMGA2: High Mobility Group AT-hook protein 2
HPDE: Human pancreatic ductal epithelial
IL20RB: Interleukin 20 receptor subunit beta
KEGG: Kyoto Encyclopedia of Genes and Genomes
MAPK: Mitogen‑activated protein kinase
NMDARs: N-methyl-D-aspartate receptors
PDAC: Pancreatic ductal adenocarcinoma
PNET: Pancreatic neuroendocrine tumorigenesis
TCA: Tricarboxylic acid
TCGA: The Cancer Genome Atlas
TNBC: Triple- Negative Breast Cancer
