# Supplementary figures and images for: Identification of GRIN2D as a novel therapeutic target in pancreatic ductal adenocarcinoma
Source: Biomark Res. 2023 Aug 8;11:74. doi: 10.1186/s40364-023-00514-4 (PMC10410818; doi:10.1186/s40364-023-00514-4)

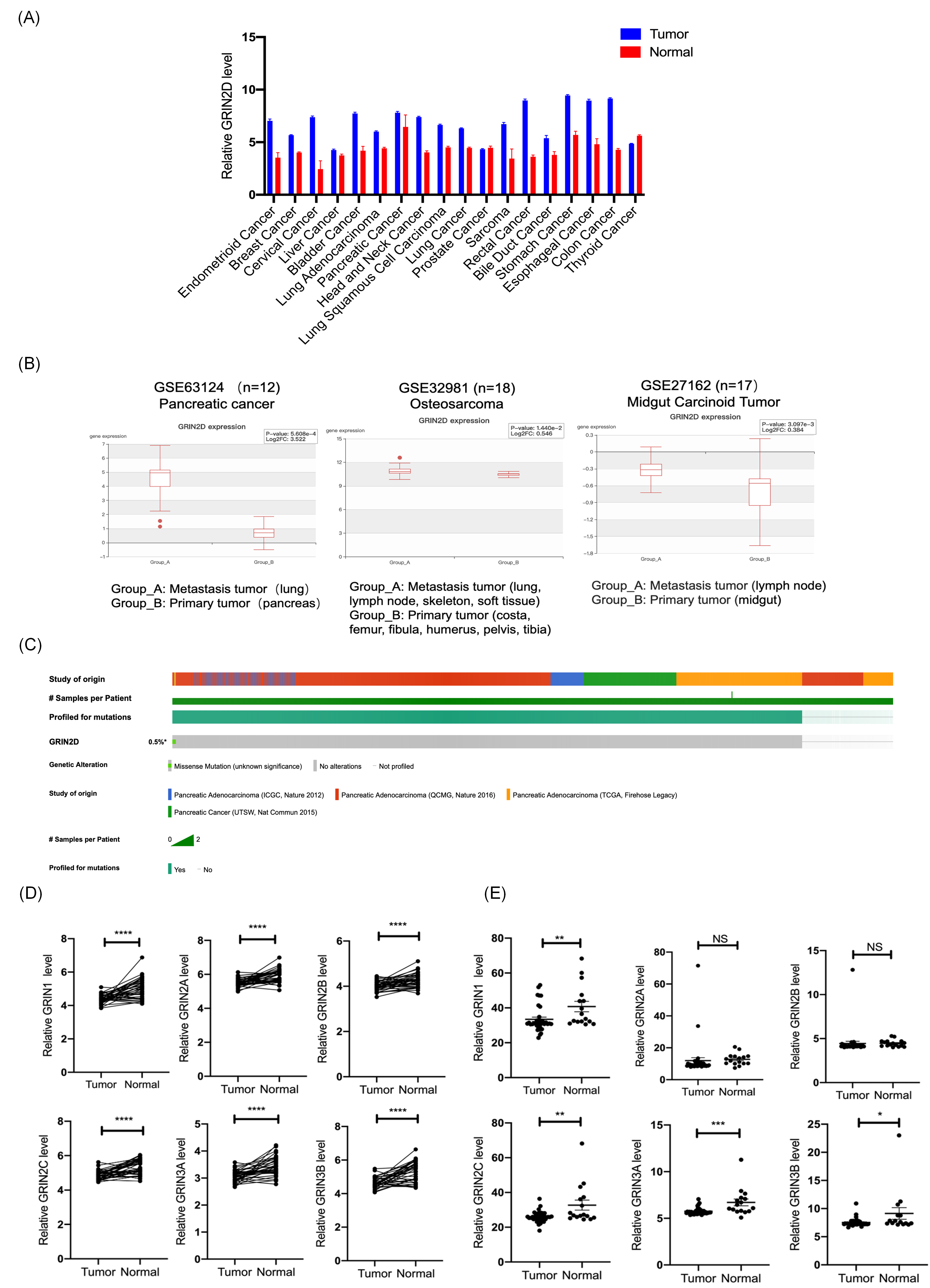

Supplement: Supplementary file 1 — Supplementary Material 1: Figure S1: (A). GRIN2D expression was upregulated in various cancers, according to samples from TCGA database. (B). Level of GRIN2D in primary tumor and metastasis tumor based on HCMDB. (C). Mutation of GRIN2D in PDAC and other cancer based on cBioPortal database. (D). Expression of NMDAR subunits GRIN2A, GRIN2B, GRIN2C, GRIN3A, and GRIN3B in PDAC primary tumors and normal tissues from patients in cohort of GSE15471. (E). Expression of NMDAR subunits GRIN2A, GRIN2B, GRIN2C, GRIN3A, and GRIN3B in PDAC primary tumors and normal tissues from patients in cohorts of GSE16515 [file 40364_2023_514_MOESM1_ESM.png]

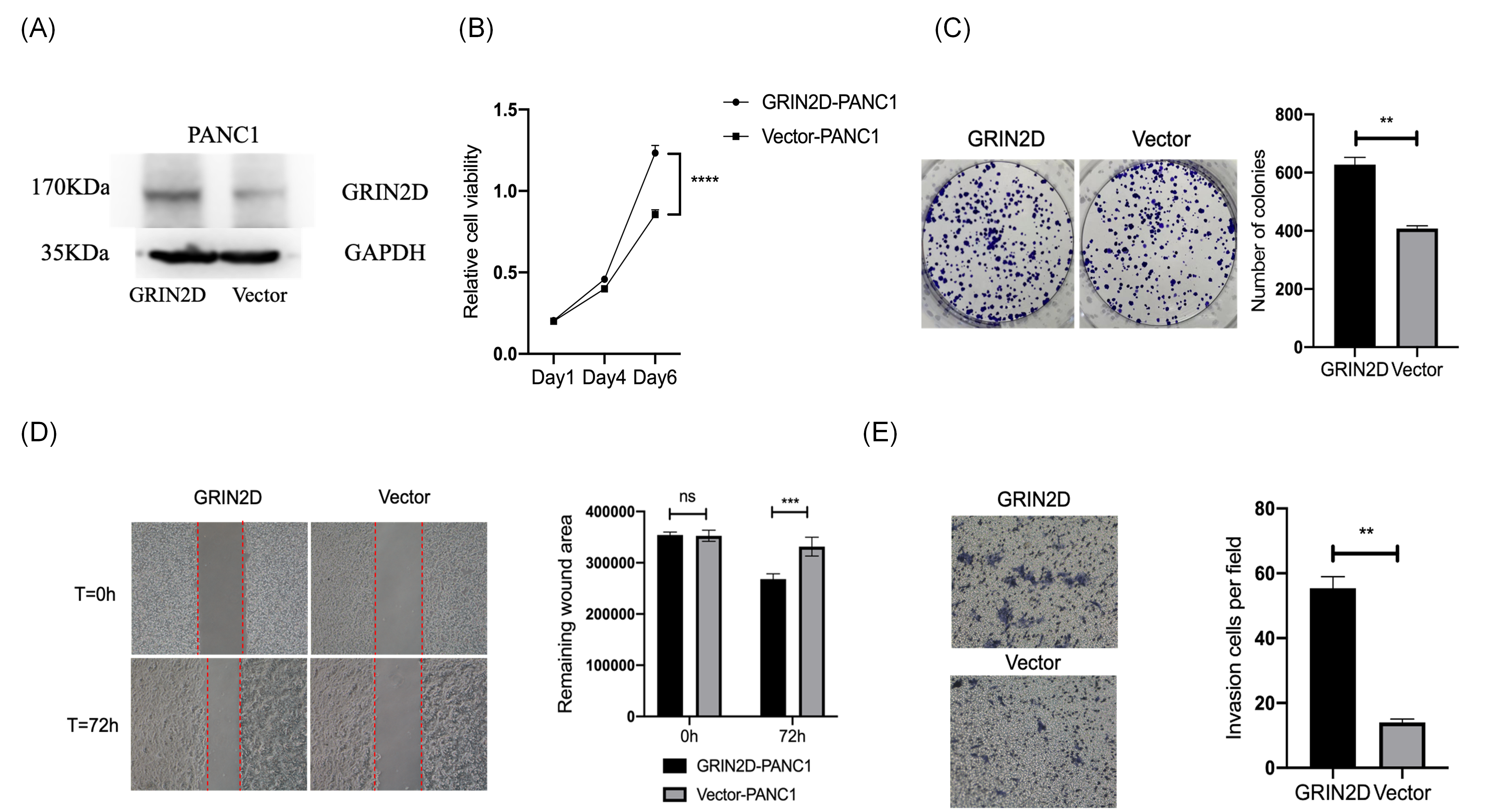

Supplement: Supplementary file 2 — Supplementary Material 2: Figure S2 (A). GRIN2D was overexpressed in PANC1 cells. (B). Overexpression of GRIN2D promoted cell growth in PANC1 cells. (C). Overexpression of GRIN2D promoted colony formation in PANC1 cells. (D). Overexpression of GRIN2D promoted cell migration in PANC1 cells. (E). Overexpression of GRIN2D promoted cell invasion in PANC1 cells. Cells in the colony formation assay and invasion assay were stained by crystal violet. Data are from at least three independent experiments. Mean ± SD. *, P < 0.05; **, P < 0.01; ***, P < 0.001 [file 40364_2023_514_MOESM2_ESM.png]

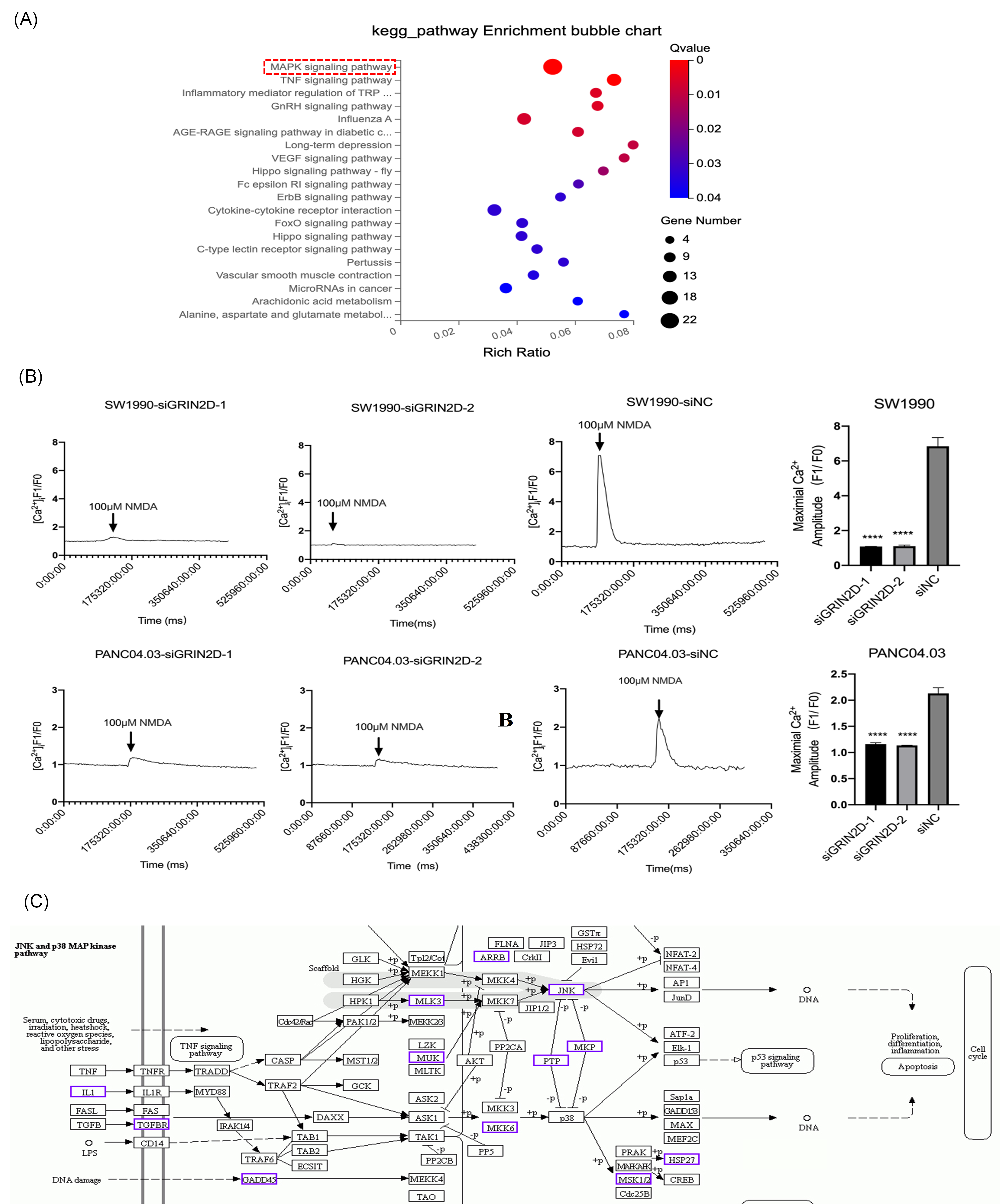

Supplement: Supplementary file 3 — Supplementary Material 3: Figure S3: (A). KEGG pathway enrichment analysis of the GRIN2D-regulated genes. (B). Calcium influx measurement after knockdown of GRIN2D in PDAC cells. (C). Map of differential genes in JNK and p38 MAPK signaling pathway [file 40364_2023_514_MOESM3_ESM.png]

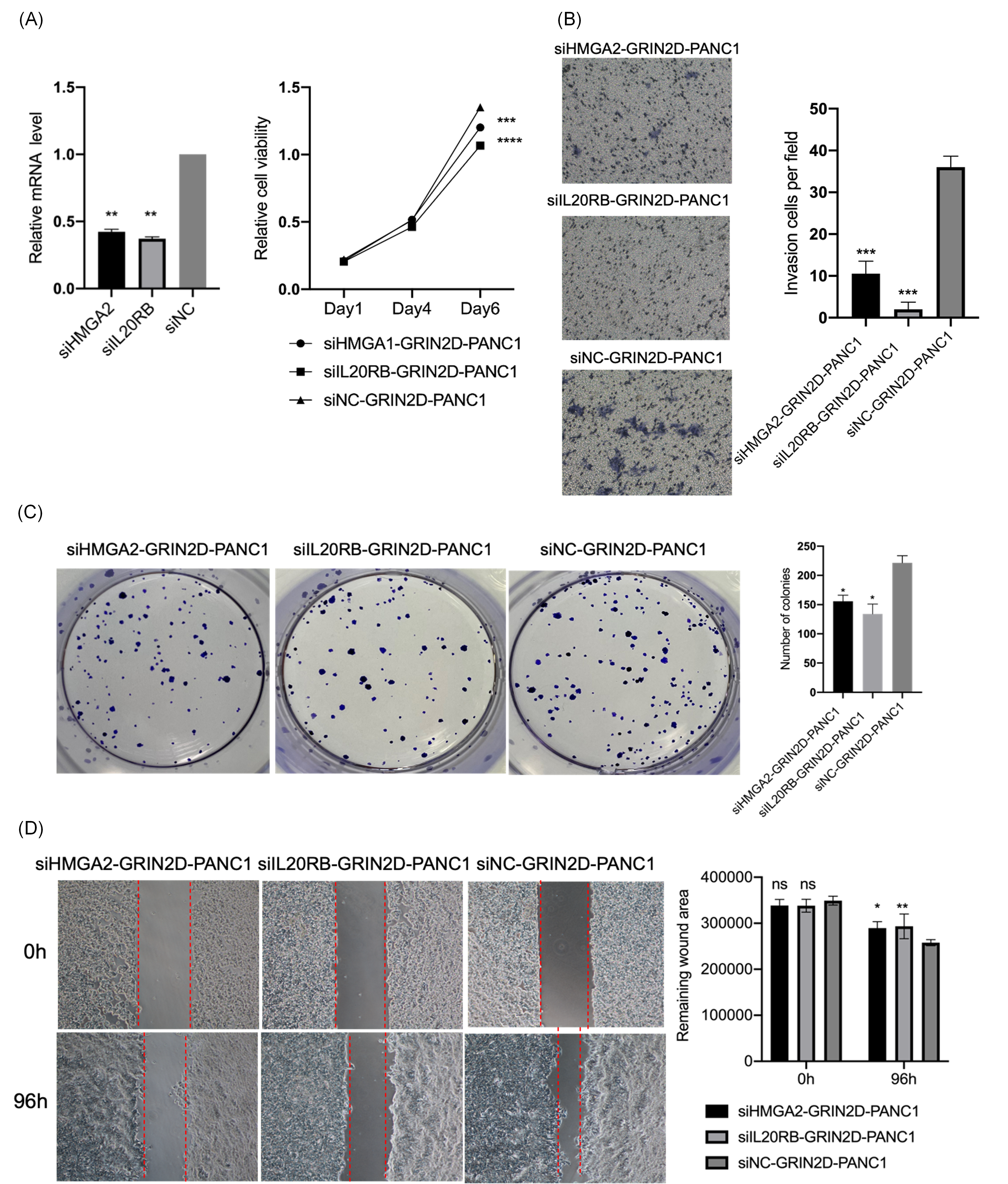

Supplement: Supplementary file 4 — Supplementary Material 4: Figure S4: (A). Knockdown of HMGA2 and IL20RB in GRIN2D- overexpressed PANC1 cells inhibited cell growth. (B). Knockdown of HMGA2 and IL20RB in GRIN2D- overexpressed PANC1 cells inhibited cell invasion. (C). Knockdown of HMGA2 and IL20RB in GRIN2D- overexpressed PANC1 cells inhibited colony formation. (D). Knockdown of HMGA2 and IL20RB in GRIN2D- overexpressed PANC1 cells inhibited cell migration. Data are from at least three independent experiments. Mean ± SD. *, P < 0.05; **, P < 0.01; ***, P < 0.001 [file 40364_2023_514_MOESM4_ESM.png]

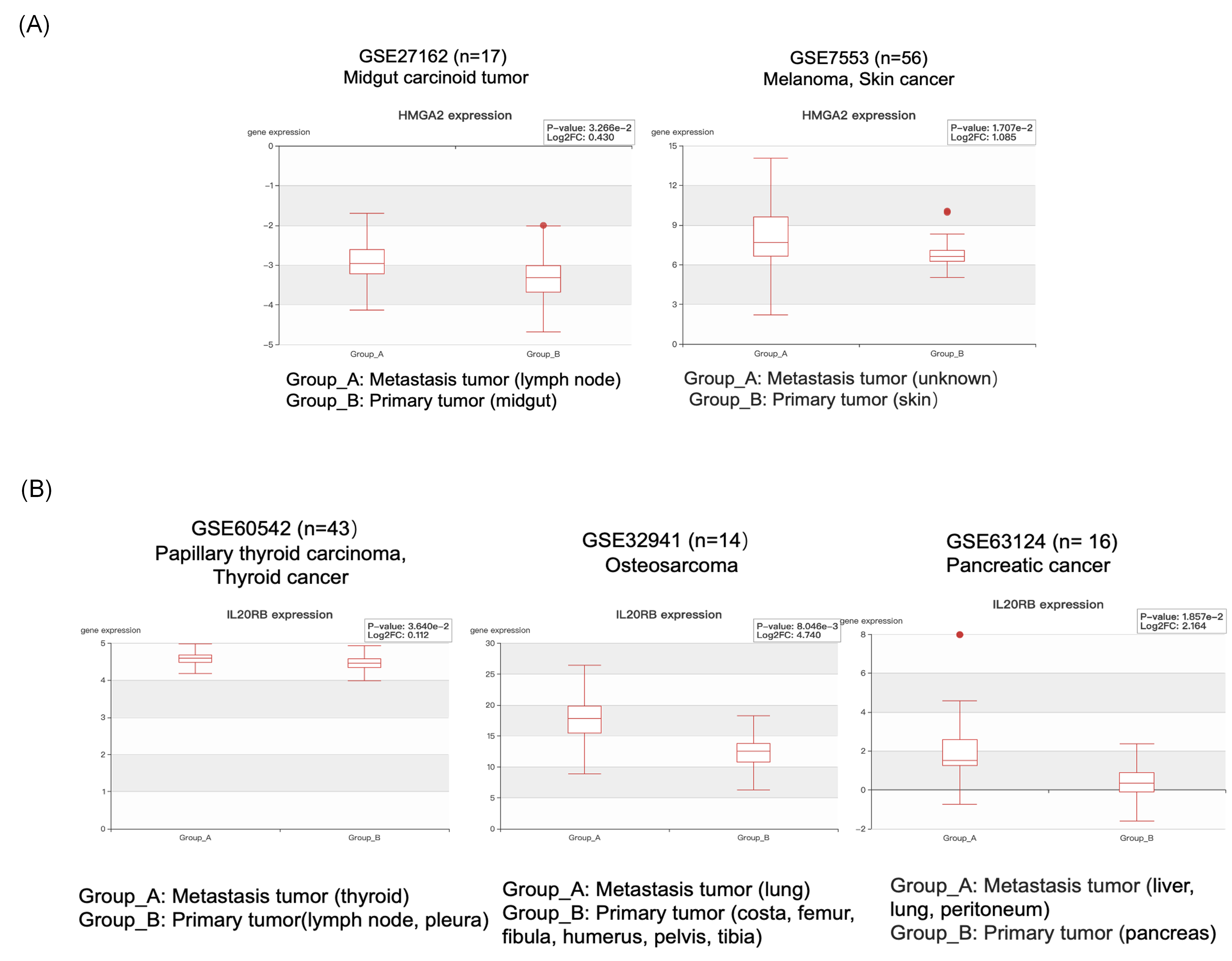

Supplement: Supplementary file 5 — Supplementary Material 5: Figure S5 (A). Level of HMGA2 in primary tumor and metastasis tumor based on HCMDB. (B). Level of IL20RB in primary tumor and metastasis tumor based on HCMDB [file 40364_2023_514_MOESM5_ESM.png]

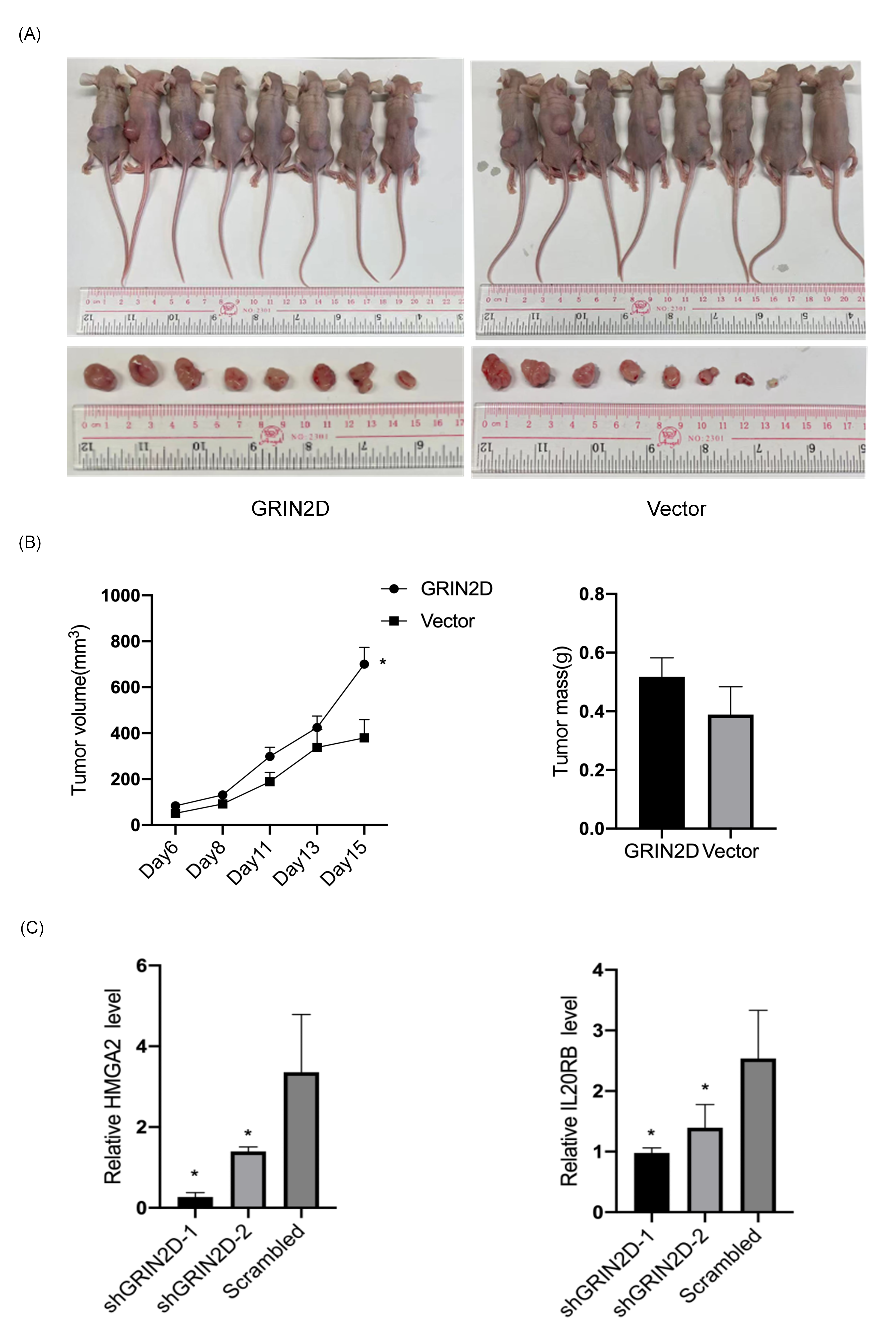

Supplement: Supplementary file 6 — Supplementary Material 6: Figure S6: (A). GRIN2D overexpression promoted tumor growth in PANC-1 cells. Photographs of mice xenograft at day 15 after overexpression of GRIN2D in PANC-1 cells. (B). Tumor volume and tumor mass of xenograft mice increased after overexpression of GRIN2D. (C). HMGA2 and IL20RB were downregulated in GRIN2D knocked down tissues. Data are from at least three independent experiments. Mean ± SD. *, P < 0.05; **, P < 0.01; ***, P < 0.001 [file 40364_2023_514_MOESM6_ESM.png]
