# Supplementary material for: Identification of GRIN2D as a novel therapeutic target in pancreatic ductal adenocarcinoma
Source: Biomark Res. 2023 Aug 8;11:74. doi: 10.1186/s40364-023-00514-4 (PMC10410818; doi:10.1186/s40364-023-00514-4)
Supplement: Supplementary file 7 — Supplementary Material 7 [file 40364_2023_514_MOESM7_ESM.docx]

**Supplementary Materials and Methods**

**Protein extraction from cells**

Culture medium was removed and the cells were washed by 1X PBS for twice. 0.25% trypsin was used for digesting cells at 37°C for 5minutes. Culture medium finalized the trypsinization, and the cells were centrifuged in 1000rpm for 5mins. After the supernatant was removed, PBS washed the cell pallet once. Cells underwent the centrifugation again in 12000g for 3mins. The supernatant was removed and cell pallet was suspended with 50uL NP40 for the cell lysis. After 20minutes incubation on ice, tubes were centrifuged in 12000g at 4°C for 20mins. The supernatant was collected and mixed with 5X loading buffer. The mixture was boiled in the metal bath at 98°C for 15 minutes. The boiled samples can be used for western blot.

**Western blot**

The equal contents of protein samples were loaded. The running procedure underwent at the 130V until the specific band were separated. The transfer procedure was under 300A for 120mins. The membrane blocking was processed in the 5% milk in 1X TBST buffer at room temperature for 2 hours. Incubation for primary antibody was conducted in 4°C overnight. Primary antibodies were all diluted with 5% milk.

The selection of secondary antibody was basing on the host of primary antibody, the incubation at the room temperature for 1 hour. The membranes were washed for three times by TBST before exposure. Exposure was performed by Chemidoc (Biorad).

**siRNA transfection**

siRNA transfection was performed by lipofectamine 3000. The day before transfection, cells were seeded in the 6 well plate with the density of 150,000 cells per well. For siRNA transfection, the siRNA was disolved to 20uM in 125uL in advance. The siRNA mix contained 5uL siRNA and 125uL, and the Lipo3000 mix contained 3.75uL lipofectamine and 125uL. Each mix should be mixed well and incubated at room temperature for 5 minutes. Then, they were mixed up together and incubated at room temperature for 30 minutes. After the incubation, the complete medium was removed then added mixture and opti-MEM, the final volume is 750uL. Cells were incubated with the transfection mix for 6-8h, the complete medium replaced the mix. After 48- 72h, the gene knocked down cells could be used for functional experiments or sample collection.

**RNA extraction**

For RNA extraction from cells: Culture medium was removed and cells were washed by PBS for two times. 500uL TRIZOL was added to 6 well dishes for collecting cells. The TRIZOL contained cells was transferred to 1.5ml tubes and left at room temperature for 5minutes. 100uL chloroform was added and shaken for 15s, the tubes were left at room temperature for 10minutes. The tubes were concentrated in 12000g at 4°C for 15minutes, the supernatant was transferred to new tubes. 250uL isopropyl alcohol was added to the new tubes and mixed with supernatant gently, the mix was left at room temperature for 10 minutes. Then, the tubes were centrifuged in 12000g at 4°C for 10minutes and discarded supernatant. The precipitation was washed by 1ml 75% ethanol, the ethanol was discarded after the centrifugation in 7500g at 4°C for 5min. The left liquid was dried for 5mins and 20uL DEPC H2O was added to dissolve the RNA. The concentration of RNA was measured by Nanodrop (Biorad).

For RNA extraction from tissues: The volume of tissues was one tens of Trizol, the tissues were grinded by homogenizer. The following steps were same as the progress of extracting RNA from cells.

**Plasmid amplification**

Plasmids were obtained from IGE BIO (China) and kept as powder. Transformation was performed firstly to amplificated the plasmids. Competent cells DH5α or STBL3 was selected basing on the recommendation of Addgene(<https://www.addgene.org>). As the plasmid powder was dissolved by 10-20 uL ddH2O, 1 uL dissolved plasmid were added to an aliquot of competent cell and mixed gently. The mixture was left on the ice for 15mins and 70s heat- shock was performed at 42°C water bath. Cooled down on ice for 10mins, 1ml sterile LB broth was added for recovering the competent cells at 37°C, 240rpm for 1h. The entire cells were spread to LB agar plate containing the appropriate antibiotic and incubated at 37°C for 16h. As the colonies was formed in appropriate density, the single colonies were picked and incubated in 4-10ml LB broth with antibiotics at 37°C 240rpm at 4- 6h. If the liquid become muddy, the bacteria liquid will dissolved by 150ml LB broth and incubated at 37°C 240rpm overnight.

**Plasmid extraction**

Bacteria was harvested by centrifugation at 6000g for 15 min at 4 C and the supernatant was removed. The procedure of plasmid extraction followed the steps of Plasmid Purification Kit (QIAGEN, USA). After the extraction, the concentration of plasmid was measured by nanodrop (BIORAD, USA).

**Lentivirus package**

The vector of plasmids for lentivirus package were pLKO.1-U6-EF1a-copGFP-T2A-puro. At the day of lentivirus package, the HEK293T which can produce the lentivirus grew to the density of 80%. There are two mix for lentivirus packaging. The DNA mix contains 3.5ug pSL3, 6.5ug pSL4, 3.5ug pSL5 and 12ug vector in 800 uL Opti-MEM. PEI mix contains 25.5uL PEI in 800uL Opti- MEM. Vortex of the two mix and incubated at room temperature for 30 minutes. The culture medium in HEK293T was replaced by the mixture and Opti- MEM, the total volume is 8ml in each 10cm dish. HEK293T cells were put in incubator for 7-8 hour and refreshed the culture medium.

After 72 hours incubation, the yellow medium was collected and centrifuged in 1000rpm for 5 minutes. The supernatant was filtrated for cell infection.

**Cell infection**

150,000 cells were seeded per well in 6 well dish in advance. As the cells were attached in the next morning, the mixture of lentivirus liquid and DMEM at the ratio of 1:2 replaced the culture medium. Polybrene was used to promote infection. After 24h, the infection medium was replaced by the fresh medium with 1 μg/ml puromycin. When the parental cells in control group were killed totally, the remaining cells in infected group were infected successfully. The infected cells can be cultured in normal condition for passage or further experiment.
